# Innate Immune Response in Kidney Ischemia/Reperfusion Injury: Potential Target for Therapy

**DOI:** 10.1155/2017/6305439

**Published:** 2017-06-06

**Authors:** Aleksandra Kezić, Natasa Stajic, Friedrich Thaiss

**Affiliations:** ^1^School of Medicine, University of Belgrade, Belgrade, Serbia; ^2^Clinic for Nephrology, Clinical Center of Serbia, Belgrade, Serbia; ^3^Institute of Mother and Child Healthcare of Serbia “Dr Vukan Čupić”, Belgrade, Serbia; ^4^Third Medical Department of Clinical Medicine, University Hospital Hamburg Eppendorf, Hamburg, Germany

## Abstract

Acute kidney injury caused by ischemia and subsequent reperfusion is associated with a high rate of mortality and morbidity. Ischemia/reperfusion injury in kidney transplantation causes delayed graft function and is associated with more frequent episodes of acute rejection and progression to chronic allograft nephropathy. Alloantigen-independent inflammation is an important process, participating in pathogenesis of injurious response, caused by ischemia and reperfusion. This innate immune response is characterized by the activity of classical cells belonging to the immune system, such as neutrophils, macrophages, dendritic cells, lymphocytes, and also tubular epithelial cells and endothelial cells. These immune cells not only participate in inflammation after ischemia exerting detrimental influence but also play a protective role in the healing response from ischemia/reperfusion injury. Delineating of complex mechanisms of their actions could be fruitful in future prevention and treatment of ischemia/reperfusion injury. Among numerous so far conducted experiments, observed immunomodulatory role of adenosine and adenosine receptor agonists in complex interactions of dendritic cells, natural killer T cells, and T regulatory cells is emphasized as promising in the treatment of kidney ischemia/reperfusion injury. Potential pharmacological approaches which decrease NF-*κ*B activity and antagonize mechanisms downstream of activated Toll-like receptors are discussed.

## 1. Introduction

Ischemia/reperfusion injury (IRI) is a leading cause of acute kidney injury (AKI), which is still associated with high morbidity, mortality, and increased costs of treatment in both adult and pediatric population [1, 2]. IRI represents an important risk factor for progression of chronic kidney disease (CKD), which is defined as abnormalities of kidney structure or function, present for more than 3 months [3, 4]. Kidneys are subjected to IRI in different medical conditions, characterized by the interruption of the renal blood flow followed by the subsequent restoration of perfusion, such as vascular and cardiac surgery, trauma, circulatory arrest with resuscitation, and kidney transplantation. IRI after kidney transplantation elicits cascades of pathophysiology processes that result in delayed graft function (DGF) and alloimmune-specific response to the graft, eventually leading to acute rejection and progression to chronic allograft nephropathy (CAN) [5].

Initial hypoxic injury with subsequent production of reactive oxygen species (ROS), due to reoxygenation, initiates events in IRI, leading to apoptosis, necrosis, and a profound inflammatory response [6]. Mitochondria, as the major site of ROS production, are subjects of potential treatment of kidney IRI using mitochondria-targeted antioxidants [7]. Besides ROS, reactive nitrogen species (RNS), such as nitric oxide, are produced in kidney IRI via the activity of inducible NO synthase (iNOS), which is considered as one of inflammatory mediators [8]. Numerous experimental studies have shown an increased iNOS activity in kidney IRI [9–11]. iNOS and a great array of proinflammatory cytokines, such as TNF-*α*, IL-1*β*, IL-6, and chemokines, are mainly elicited by the action of transcription factors NF-*κ*B . This inflammatory response is sterile but shares many similarities with the immune response and inflammation provoked by pathogens [12]. Evidence supporting immunological mechanisms involved in pathogenesis of IRI has accrued in recent years, pointing to the activation of both innate and acquired immune response [13–17]. In early phase of IRI, inflammation is alloantigen independent and is characterized by activation of not just only classical cells belonging to what we call the immune system, such as macrophages, dendritic cells, and lymphocytes, but also resident renal cells, such as endothelial cells and tubular epithelial cells, which are extremely sensitive to oxidative stress. Interplay and time-dependent changes between proinflammatory and anti-inflammatory mediators, secreted by kidney resident cells and recruited inflammatory cells, determine the faith of renal injury, namely, tissue repair or progression to CKD [18, 19].

Numerous studies conducted so far do not give precise answers on how to treat or prevent kidney IRI implying inflammation and mediators of inflammation as potential target for therapy. In this article, we will discuss some features of the innate immune response in kidney IRI, emphasizing the optimal and potentially the most successful approach to treat kidney IRI based on so far achieved results and knowledge.

## 2. The Role of Endothelial and Tubular Epithelial Cells in Inflammation Provoked by IRI

**Figure 1:**
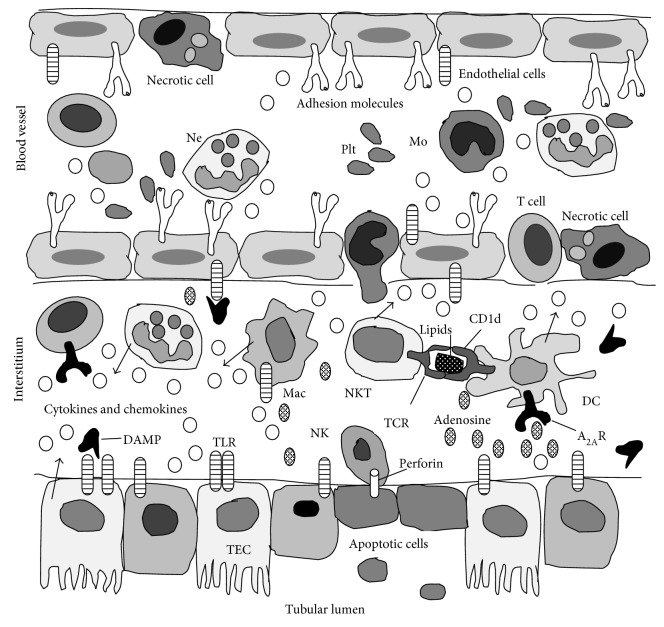
Inflammation in kidney ischemia/reperfusion injury. During reperfusion, immune cells increase their adhesiveness and adhere to activated endothelium and some of them migrate into the interstitium, continuing the inflammation together with the resident renal immune cells, by secretion of numerous cytokines, chemokines, oxygen-free radicals, complement, and other mediators. Among other mediators there is adenosine that downregulates inflammation, acting via A_2A_R expressed on dendritic cells and T cells. A_2A_R: adenosine receptor; CD1d: glycoprotein presenting lipids and glycolipids to NKT; DAMP: danger-associated molecular patterns; DC: dendritic cell; Mac: macrophage; Mo: monocyte; Ne: neutrophil; NK: natural killer cell; NKT: natural killer T cell; Plt: platelet; TCR: T cell receptor; TEC: tubular epithelial cell; TLR: Toll-like receptor.

Kidney ischemia affects primarily the structure and function of tubular epithelial cells, leading to apoptosis and/or necrosis, followed by interstitial inflammation and microvasculopathy during reperfusion, that accentuate the injury. At the beginning of reperfusion, a cortical circulation is established, but in the outer part of medulla, hypoperfusion still persists and is characterized by the congestion of peritubular capillaries that show swelling of endothelial cells, increased paracellular permeability, and increased expression of adhesive molecules, such as intercellular adhesion molecule 1 (ICAM-1) and E- and P-selectins, and leukocyte accumulation with platelet aggregates [15, 20–22] (Figure 1). This *no reflow* phenomena are caused by endothelial dysfunction that is characterized by increased vasoconstriction and decreased synthesis of vasodilatatory substances like NO [23, 24]. ICAM-1 expression increases by 1 hour after IRI and accelerates neutrophil adhesion and migration in perivascular tissue [20]. Endothelial cells also increase the expression of chemokine fractalkine (CX3CL1) that is a ligand for receptor CX3CR1, significantly expressed on macrophage membrane and important for their migration from the blood vessels into the interstitial tissue [25].

Ischemia provokes a massive release of compounds from the damaged tissue called danger-associated molecular patterns (DAMPs), such as hyaluronic acid, fibronectin, heat shock proteins (HSP), and DNA which activate Toll-like receptors (TLR) 2, 4, and 5, the evolutionary conserved family of transmembrane receptors that are a type of the pattern recognition receptor (PRR) [26]. The activation of PRR may induce both death signaling pathway and the production of proinflammatory cytokines [27]. When engaged, TLR elicit the production of a great array of proinflammatory cytokines and chemokines, such as TNF-*α*, IL-1*β*, IL-6, CCL2, MIP-2, and chemokines produced by keratinocytes (KC), further accompanied by neutrophil and macrophage infiltration [16, 17, 28, 29]. Transduction of signals after TLR activation depends on several adaptive proteins, among which MyD88 is the most important, resulting in activation of transcription factor NF-*κ*B, with subsequent production of proinflammatory cytokines and chemokines [28, 29]. Besides macrophages and dendritic cells, tubular epithelial cells constitutively express TLR2 and TLR4, and this expression is increased during kidney IRI [28–30] (Figure 1). It has been shown that renal TLR2 signaling contributes to the acute kidney injury and inflammation during IRI [31]. TLR4 deficiency on kidney parenchyma cells prevented the increase of chemokine and proinflammatory cytokine production and neutrophil and macrophage accumulation in experimental kidney IRI model [29]. According to those experimental results, one can suppose that inhibitors of TLR signaling could be effective in preventing sterile inflammation induced by IRI. Recently, it has been shown that mice subjected to kidney IRI and treated with TJ-M2010-2, which inhibited MyD88 homodimerization, had better survival rate than nontreated animals [32]. Additionally, treatment with TJ-M2010-2 markedly attenuated inflammatory response and ameliorated tubular interstitial fibrosis in mice subjected to kidney IRI [32]. Human studies implicating TLR in ischemic AKI have not been conducted so far, but there are some studies investigating TLR4 antagonist TAK-242 in other clinical conditions, such as sepsis and shock [33]. Based on experimental studies confirming TLR role in kidney IRI, translational approaches using TLR antagonists in kidney IRI remain promising.

Another role of tubular epithelial cells in innate immune response is represented by the modulation of complement system. A unique feature of complement system in kidney IRI is that activation occurs predominantly by the alternative pathway [34]. The activation of complement by an alternative pathway is a prerequisite for the production of CXC chemokines. Tubular epithelial cells express a complement inhibitor called Crry on basolateral membrane. During IRI, there is Crry redistribution that allows C3 deposition and complement activation by the alternate pathway [35]. These events elicit production of keratinocyte-derived chemokine (KC or CXCL1) and MIP-2 (CXCL2) that attract neutrophils and macrophages to the kidney tissue. Selective inhibition of the alternative pathway protects the kidney from ischemic injury [36, 37].

## 3. Neutrophils, Macrophages, and Dendritic Cells in Kidney IRI

As early as 30 minutes after the reperfusion, neutrophils attach to activated endothelium in peritubular capillaries and accumulate in kidney interstitium, both in animal models and in human AKI [6]. Neutrophil-endothelial adhesion is mediated by lymphocyte function-associated antigen- (LFA-) 1 (CD11a/CD18) that is a counterreceptor for ICAM-1 (CD54). By producing reactive oxygen species, myeloperoxidase and cytokines such as IL-17, neutrophils aggravate kidney injury [38]. Neutrophil-depleted mice, mice treated by anti ICAM-1 antibody, and ICAM-1 knockout mice were protected against kidney IRI [14, 39]. The detrimental role of neutrophils in pathogenesis of acute kidney injury was shown in experimental mice models, but was not confirmed completely in humans. A phase I of human trial investigating the use of anti-ICAM-1 monoclonal antibody in 18 recipients of cadaveric renal transplants showed lower rates of DGF [40], but a randomized controlled trial failed to show beneficial effects of ICAM-1 blocking on the rate of DGF [41].

Macrophages infiltrate the kidney just after neutrophils transmigrate into it. Signal transduction pathways elicited by activation of CCR2 and CX3CR1 seem critical in macrophage migration process, although CCR1 pathway is also involved [42–44]. CCR2-deficient mice were protected from kidney IRI and showed reduced kidney macrophage infiltration [44]. CX3CR1 deficiency and treatment with a CX3CR1 blocking antibody protected kidneys from IRI [25]. In the early phase of innate immune response, macrophages and dendritic cells contribute to tissue damage by producing a great array of chemokines and proinflammatory cytokines (TNF-*α*, IL-6, CCL2, CCL5, IL-12, and IL-23) [45–47]. In addition, dendritic cells presenting glycolipids by CD1d molecules to natural killer T cells (NKT), and making contact with NKT by CD40-CD40L interaction, activate NKT to produce IFN-*γ*, a powerful stimulator of macrophages, thereby amplifying the innate immune response [47–49] (Figure 1). Macrophages and dendritic cells are a heterogeneous population of cells that are capable of inducing “sterile” inflammation following reperfusion. CD11c^+^ MHCII^+^ dendritic cells represent the most abundant leukocytes subset in kidneys [50]. Their increased number has been found in transplanted kidneys in rat experimental model and human transplanted kidney specimens, confirming the role of dendritic cells in DGF and acute rejection [51, 52]. Precursors of dendritic cells and macrophages are blood monocytes, which patrol and monitor the endothelium. “Resident” monocytes characterized as CCR2^−^CX3CR1^high^GR-1^−^Ly6C^−^ migrate into the tissue where they differentiate into resident macrophages and dendritic cells, whereas “inflammatory” monocytes defined as CCR2^+^CX3CR1^low^GR-1^+^Ly6C^high^ are recruited to injured, inflamed tissue and differentiate into active, “inflamed” macrophages and dendritic cells [48, 53, 54]. This differentiation is influenced by prevailing local microenvironment, for example, cytokine milieu. TNF-*α*, IL-4, and IL-15 skew monocyte differentiation towards dendritic cells, whereas IFN-*γ* and IL-6 direct monocyte differentiation towards macrophages [48, 55–58]. “Inflamed” macrophages, called M1 type, produce NO and secrete proinflammatory cytokines like IL-1*α*, IL-6, TNF-*α*, and IL-12, thereby causing tissue damage [59, 60]. Also, the macrophage phenotype switch parallels the course of inflammation and repair process. While M1-type macrophages play proinflammatory role, M2-type macrophages are generally believed to play prorepair role, have high phagocytosis activity, drive Th2 immune response, and are generated when monocytes are exposed to IL-4, IL-13, immune complexes, or IL-10 [61–64].

Depletion of kidney and spleen macrophages using clodronate liposomes before IRI prevents AKI, while adoptive transfer of macrophages restored the AKI response [65, 66]. On the contrary, in the recovery phase of IRI, macrophages appear essential for appropriate tissue repair, and using clodronate liposomes in that phase is deleterious for the kidney tissue [65, 66]. Those data are in line with the protective role of M2-type macrophages in appropriate resolution and tissue repair. Also, treatment of mice that are transgenic for human diphtheria toxin receptor (DTR) by diphtheria toxin (DT) prior to kidney IRI kills primarily CD11c^+^ DCs, leading to significantly lower rise in plasma creatinine in those animals, and less tubular cell necrosis, in comparison to DT negative or mutant DT-treated wild-type control mice [48]. These experiments provide strong evidence for the participation of dendritic cells in early response in kidney IRI. Therapeutic interventions in ischemic AKI targeting dendritic cells could be promising in the future. In this regard, Li and coworkers found that mice deficient in the adenosine receptor A_2A_R only on CD11c^+^ DCs had accentuated inflammation and significant worsening of kidney function, while treatment by selective A_2A_R agonist ATL313 protected the kidneys [67]. Adenosine mediates anti-inflammatory action in inflamed tissues [68]. The hypoxia is associated with a high levels of extracellular adenosine that activate Gs-coupled A2A and/or A2B (A2BR) adenosine receptors on the surface of surrounding immune cells leading to increased intracellular cAMP concentration [69]. One of possible mechanism implicated in immunosuppressive effect of A_2A_R activation is inhibition of NF-*κ*B pathway downstream of immunoreceptors by elevated intracellular cAMP [70].

In experiments conducted in A_2A_R-deficient mice, it has been shown that increased transcription of proinflammatory cytokines was associated with enhanced activity of the NF-kappaB [71]. A_2A_R inhibits TLR-induced transcription of proinflammatory cytokines in vivo. Also, A_2A_R downregulate the cytokine and chemokine transcripts in kidney and liver tissues in ischemia-reperfusion injury [72, 73]. The administration of DCs treated ex vivo with an A_2A_R agonist protected the kidneys of WT mice in IRI [67]. This treatment was effective in the prevention of IRI, but there were no effects if the treatment started 24 hours after the IRI induction. A key mechanism, by which adenosine-mediated DC cell therapy is successful in AKI, appears to be suppression of IFN-*γ* production by NKT and increased production of anti-inflammatory cytokine IL-10 [67]. Also, the role of CD4^+^ T cells has been implicated in the protective effects of A_2A_R agonist ATL146e on ischemia-reperfusion damage in the kidney [74]. The beneficial effect following A_2A_R activation on CD4^+^ T cells resulted in decreased IFN-*γ* production and neutrophil recruitment.

Another approach is the development of sphingosine 1-phosphate-3 receptor (S1P3R) antagonist. Sphingosine 1-phosphate (S1P) is a major sphingolipid metabolite that is the ligand for a family of five G-protein-coupled receptors (S1PRs) with diverse cellular signaling responses [75]. The different biological processes, such as immune response, cell migration, and angiogenesis, depend on the pattern of S1PR expression and the different downstream signaling molecules [75]. Depending on the DC maturation status in mouse, the different expression profile of S1PRs can be found including S1P1 and S1P3. Mature DCs express higher levels of S1P3 mRNA compared with immature DCs [76]. Via S1P3 but not S1P1, S1P regulates migration and endocytosis of mature murine DCs [76]. The other authors accentuate the significance of both S1P3 and S1P1 in migration of mature DCs to S1P [77]. Mice that had S1P3-deficient dendritic cells were protected from IRI [78]. These S1P3-deficient DCs displayed an immature phenotype and activated the Th2/IL-4 pathway in NKT cells. If S1P3-deficient DCs were administered to mice 7 days prior to or 3 h after IRI induction, those animals were protected from IRI [78]. These findings support the development of selective S1P3 antagonists that can be used for tolerating DCs in cell-based therapy or in systemic administration for the prevention and treatment of IRI. The mechanism of S1P3R blockade is a decreased activity of NF-*κ*B. Dendritic cells from S1pr3^−^/^−^ mice have significantly lower translocation of NF-*κ*B to the nucleus. DC maturation and immunostimulatory activity, that is, expression of costimulatory molecules (CD40/80/86) are driven by NF-*κ*B-dependent gene transcription [79, 80]. As a result, S1PR3^−^/^−^ dendritic cells are unable to induce an inflammatory cytokine response.

## 4. Natural Killer T Cells and T Lymphocytes in Kidney IRI

Natural killer T cells (NKT) with dendritic cells are considered the most important link between the innate and acquired immunity. NKT represent a unique T cell population, possessing a natural killer (NK) cell-associated marker NK1.1 (CD161), and a T cell receptor (TCR), that does not recognize peptides presented by MHC I or MHC II molecules [81]. These cells recognize lipids and glycolipids presented by CD1d molecules [82]. The best-known subset of CD1d-dependent NKT cells is type I or invariant NKT (iNKT) cells, known for expressing invariant T-cell receptor (TCR) *α* chain [83]. After the recognition of those molecules, iNKT produce a significant amount of proinflammatory cytokines of Th1 type (IFN-*γ*, TNF-*α*) and Th2 type (IL-4, IL-13) [83, 84]. The infiltration of these cells in the kidney starts even 30 min after the IRI induction, and maximal IFN-*γ* production is achieved 3 h following reperfusion [47]. iNKT directly regulate and amplify the function of dendritic cells and indirectly the function of T cells, thereby linking the innate and acquired immunity. The importance of iNKT in pathogenesis of IRI is corroborated by the fact that the blockade of iNKT using anti-CD-1d antibody or anti-NK1.1 antibody prevents AKI following IRI [47].

Numerous studies have shown the importance of CD4^+^ T cells functioning in the early phase of IRI with other cells in the innate immune response. Kinetic analysis showed significant infiltration of CD4^+^ T cells in kidneys even 1 hour after the IRI induction [85]. Mice deficient in T cells (“*nu/nu*” mice) are protected from kidney IRI, but the injury is induced if the adoptive transfer of CD4^+^ T cells has been made to those mice [85]. Interestingly, experiments with *Rag1^−/−^* mice, which lack both T and B cells, found no protection from AKI induced by ischemia [86]. These observations suggest either the important role of NK cells or another type of cells belonging to the innate immunity in pathogenesis of kidney IRI, or that complex interactions between B and T cells may occur in kidney IRI, also implying protective roles of some immune cells in IRI. NK cell depletion in wild-type C57BL/6 mice was protective in kidney IRI implying the important role of NK cells [87]. It has been shown that NK cells can directly kill tubular epithelial cells, contributing substantially to kidney IRI (Figure 1) [87].

T cells migrate in kidney tissue by CD28-B7-1 interaction. Endothelial B7-1 expression on ascendant parts of vasa recta was found in human kidneys after ischemia [88]. The treatment of animals with monoclonal antibodies to B7-1 reduced kidney injury caused by ischemia [89]. Flow cytometry of kidney lymphocytes, detected 24 hours after the induced IRI, detected an increased number of T cells producing IFN-*γ* and TNF-*α* [90]. The adoptive transfer of CD4^+^ T cells deficient in CD28 expression or IFN-*γ* secretion has failed to reconstitute kidney IRI in *nu/nu* [85]. These results implicate the significance of Th1 cells for pathogenesis of IRI. The importance of Th1 cells in kidney IRI was confirmed by CCR5 blockade, the receptor for chemokine CCL5, which is predominantly expressed on Th1 cells [91]. The blockade of CCR5 by neutralizing the antibody protected mice from kidney IRI [91]. CXCR3 is also expressed on Th1 cells. CXCR3 “*knock-out*” mice subjected to kidney IRI had better survival rate, better kidney function and decreased tubular necrosis, and inflammatory cell infiltrate, compared to wild-type mice [92]. Also, the detrimental role of Th17 cells in pathogenesis of kidney IRI has recently been confirmed [93, 94]. The intricate role of NF-*κ*B has been implicated in the induction of Th17 lineage. The differentiation of Th17 cell is driven by NF-*κ*B pathway activation through c-Rel induction of RAR-related orphan receptor gamma (ROR*γ*1 and ROR*γ*2) expression [95, 96]. Other studies showed anti-inflammatory role for NF-*κ*B kinases IKK2 through the inhibition of “classical” pathway activation [93, 97, 98]. The deletion of the IKK2 or NEMO in lymphocytes caused more tubular damage and Th17 cell infiltration, comparing to CD4cre mice after renal IRI induction [93]. It has been proposed that noncanonical NF-*κ*B activation directs the development of Th17 cell immune response that is inhibited by the IKK2-mediated canonical NF-*κ*B pathway [93].

On the contrary, Th2 cells and T regulatory (Treg) cells seem to be protective in IRI. Mice which lack of IL-4, mediator of Th2 response, have significantly greater kidney ischemic injury, compared to wild type, and pointing to protective role of Th2 cells [99]. Treg cells are characterized by CD25 expression and the activity of transcription factor called “*forkhead box P3*” (FoxP3). Treg cells produce anti-inflammatory cytokines IL-10 and TGF-*β* and modulate the immune response. IKK2 activity and NF-*κ*B pathway play a critical role in peripheral Treg cell proliferation and maturation [100]. In the previously mentioned experimental model of kidney IRI in mice which had deleted IKK2 or NEMO in lymphocytes (CD4xIKK2^Δ^ and CD4xNEMO^Δ^ mice), Treg cells were significantly reduced [93]. It has been shown that the depletion of CD4^+^CD25^+^ Treg cells by anti-CD25 antibody did not influence the early phase of IRI, but caused more necrosis, measured on day 3 after the induced IRI, suggesting a beneficial role of Treg cells in the recovery phase [101]. Treg cells become a promising candidate for cell therapy, since these cells are effective even when administered 24 hours after the ischemic insult [102], while already mentioned therapy by adenosine modified dendritic cells is effective if it is administered up to 6 hours after the beginning of ischemia.

This therapeutic option by Treg cells is limited by difficulties arising from isolating and expanding desirable Treg cells. Some investigators used an alternative approach with a pharmacologic agent to stimulate Treg cells [103]. Pretreatment of mice with the sphingosine N,N-dimethylsphingosine (DMS) increases both tissue-infiltrating T effectors (CD4^+^Foxp3^−^) and Treg (CD4^+^Foxp3^+^) cells and ameliorates IRI in experimental model of renal IRI. The administration of the anti-CTLA-4 or anti-CD25 monoclonal antibodies, which are Treg cells suppressing agents, abolished DMS protective effect suggesting a key role of Treg cells in DMS-induced renoprotection. These results point to DMS as the Treg cells recruiting drug for the treatment and prevention of kidney ischemic injury. Ex vivo expanded human Treg cells had beneficial effects in a model of transplant atherosclerosis, indicating that this way of treatment is feasible in the setting of kidney IRI [104]. Another approach to enhance Treg cell function in kidney IRI is increasing of FoxP3 expression, the key transcription factor for Treg cell differentiation, by using pharmacological inhibitors of histone/protein deacetylases, which has been effective in treating experimentally induced inflammatory bowel disease and in improving cardiac and islet graft survival in mouse transplantation models [105]. The previously mentioned immunosuppressive effect of dendritic A_2A_R stimulation has been approved in population of Treg cells. A_2A_R stimulation expanded CD4(^+^) CD25(^+^) FoxP3(^+^) cells and increased their immunosuppressive activity [106]. This fact potentiates the role of adenosine, that is, A_2A_R agonists in T cell and DC immunomodulation and IRI treatment. Summarized potential pharmacological approaches in kidney IRI are depicted in Table 1.

## 5. The Role of NF-*κ*B in Kidney IRI

So far, observed data implicate NF-*κ*B as one of the key players in pathogenesis of IRI [107–110]. A large body of evidence implicates NF-*κ*B in the production of reactive oxygen species (ROS), chemokines, cytokines, and in the control of pro- and antiapoptotic signaling, seemingly very important in the pathogenesis of IRI [107, 111–113]. NF-*κ*B functions as a crucial transcription factor in both tubular epithelial cells and inflammatory cells, linking the coordinated inflammatory and cell death signaling pathways proposed in the concept of necroinflammation. It has been shown that NF-*κ*B activation in renal tubular epithelial cells aggravated tubular injury and exacerbated inflammation in a mouse model of kidney IRI [114]. It encouraged investigators to search for NF-*κ*B-based treatments of IRI.

The inhibition of multiple steps in the NF-*κ*B activity tested in animal experiments, mainly using gene therapy (small interfering RNA and olygodeoxinucleotides), resulted in preservation of kidney function after the induced ischemia [107, 110, 115]. It has been shown that proteasome inhibition using lactacystin ameliorated renal dysfunction in kidney IRI [116]. Also, the derivative of lactacystin PS519 ameliorated cerebral ischemia [117]. This effect is attributed to the inhibition of NF-*κ*B activation via decreased degradation of I*κ*B*α*, inhibitory protein of NF-*κ*B. Another approach would be the use of synthetic IKK inhibitors. Systemic inhibition of IKK2 using KINK-1 reduced BUN and serum creatinine, but “paradoxically” increased the percentage of infiltrating Th17 cells in mouse model of kidney IRI [93]. Therefore, it could be concluded that systemic IKK inhibition has more beneficial effects on tubular epithelial cells than on infiltrating lymphocytes. Dual nature of systemic use of IKK2 inhibition has also been confirmed in experimental model of rapidly progressive glomerulonephritis [118]. Therapeutic IKK2 inhibition even produced proinflammatory effects, because suppressed Treg cells' function prevailed over the inhibited DC maturation, arguing against using IKK2 inhibitors in glomerulonephritis and possibly other immune-mediated renal diseases [118].

## 6. Conclusion

All the presented data accentuate the influence of the innate immune response in pathogenesis of IRI, giving potential options in therapeutic approach. However, to our knowledge, all these approaches are still waiting to be translated into larger clinical trials investigating IRI.

There are several questions that should be answered, such as different roles of inflammatory cells depending on the phase of IRI and their participation in progression of AKI to CKD, or why studies in rodents targeting some inflammatory pathways have shown protection while clinical studies targeting the same pathways have failed. Also, the discovery of underlying mechanisms linking ischemic graft injury and acute rejection and how they could be modified with novel therapeutics would be of great clinical importance.

## Figures and Tables

**Table 1:** Potential pharmacological approaches targeting immunological mechanisms in ischemia/reperfusion injury.

Target	Mechanism	Drug
TLR	Antagonists of TLR	TAK242 TJ-M2010-2

NF-*κ*B	Decreased degradation of NF-*κ*B inhibitory protein I*κ*B*α*	Proteasome inhibitor (Lactacystin, PS519)

Dendritic cells	A_2A_R agonists	ATL313 ATL146e

Treg cells	Expansion of Treg cells	Sphingosine N,N-dimethylsphingosine Histone/protein deacetylases inhibitors A2aR agonists
